# Emergency department visits and hospitalizations among hemodialysis patients by day of the week and dialysis schedule in the United States

**DOI:** 10.1371/journal.pone.0220966

**Published:** 2019-08-15

**Authors:** Sai Zhang, Hal Morgenstern, Patrick Albertus, Brahmajee K. Nallamothu, Kevin He, Rajiv Saran

**Affiliations:** 1 Kidney Epidemiology and Cost Center, School of Public Health, University of Michigan, Ann Arbor, MI, United States of America; 2 Department of Epidemiology, School of Public Health, University of Michigan, Ann Arbor, MI, United States of America; 3 Department of Environmental Health Sciences, School of Public Health, University of Michigan, Ann Arbor, MI, United States of America; 4 Department of Urology, Medical School, University of Michigan, Ann Arbor, MI, United States of America; 5 Institute for Healthcare Policy and Innovation, University of Michigan, Ann Arbor, MI, United States of America; 6 Department of Internal Medicine, Medical School, University of Michigan, Ann Arbor, MI, United States of America; 7 College of Medicine, University of Cincinnati, Cincinnati, OH, United States of America; 8 Ann Arbor VA Center for Clinical Management and Research and University of Michigan Health System, Ann Arbor, Michigan, United States of America; 9 Department of Internal Medicine, Michigan Integrated Center for Health Analytics and Medical Prediction, University of Michigan, Ann Arbor, Michigan, United States of America; 10 Department of Biostatistics, University of Michigan, Ann Arbor, MI, United States of America; University of Liège, BELGIUM

## Abstract

**Background and objective:**

Previous reports indicated that patients on thrice-weekly hemodialysis (HD) had higher mortality rates after the 3-day interdialytic interval. However, day-of-the-week patterns of emergency department (ED) visits and hospitalizations remain under-investigated.

**Methods:**

We conducted a retrospective cohort study of HD patients on thrice-weekly dialysis, using 2013 data from the United States Renal Data System (USRDS). We estimated crude incidence rates of ED visits and hospitalizations by day of the week and dialysis schedule (Monday, Wednesday, Friday or Tuesday, Thursday, Saturday). Using Poisson regression, we estimated case-mix adjusted rate ratios of all-cause ED visits and hospitalizations, and adjusted rates of cause-specific ED visits and hospitalizations.

**Results:**

We identified 241,093 eligible HD patients in 2013, who had 514,773 ED visits and 301,674 hospitalizations that year. Three distinct but related patterns of outcome events were observed. Crude and adjusted incidence rates of all-cause, cardiovascular, and infection-related ED visits and hospitalizations, but not vascular-access-related events, were higher on all three HD treatment days (“dialysis-day effect”). Rates for ED visits and hospitalizations were lower on weekends than weekdays, rising appreciably from Sunday to Monday for both dialysis schedules (“post-weekend effect”); and rates were highest after the long 3-day interval between dialysis sessions for both dialysis schedules (“interdialytic-gap effect”). In contrast, rates of hospitalizations not preceded by an ED visit were nearly the same Monday through Friday and lower on weekends for both dialysis schedules.

**Conclusions:**

Higher rates of ED visits and hospitalizations on the days of HD sessions and early in the week are a public-health concern that should stimulate research to explain these patterns and reduce the excessive morbidity and associated costs among patients on thrice-weekly HD, while improving quality of care and patient experience with dialysis.

## Introduction

Hemodialysis (HD) is the most common form of renal-replacement therapy in the United States (US). The majority of patients with end-stage renal disease (ESRD) receive outpatient HD three times per week on either Monday/Wednesday/Friday (MWF) or Tuesday/Thursday/Saturday (TTS). Thrice-weekly HD treatments became entrenched in practice primarily because of logistic, patient, or provider convenience and economic concerns rather than from empirical evidence [1]. A few investigators have found that all-cause mortality and cardiovascular disease (CVD) mortality were highest on the day following the 3-day interdialytic interval [2–4], with one reporting an approximately 40% higher mortality [3]. While a few studies have shown an increase in hospital admissions following the interdialytic interval for patients on thrice-weekly hemodialysis [2,4,5], the weekly patterns of emergency department (ED) visits with or without hospitalization have not received much attention, even though it is well-recognized that ESRD patients have ED utilization rates about six times the national average for US adults, and those rates are even higher among newly diagnosed ESRD patients [6].

The most prevalent renal replacement therapy among those with ESRD in the form of the unequivocally ‘non-physiological’ thrice-weekly HD [7–9], is likely insufficient to optimally correct fluid overload and often necessitates high ultrafiltration rates, predisposing patients to intradialytic hemodynamic instability [10,11]. We hypothesized that given the high frequency of intradialytic complications, higher rates of ED visits and hospitalization would not only occur early in the week (similar to mortality) but also on the days of HD sessions. In this study, we present a detailed investigation of ED visits and hospital admission rates by day of the week and dialysis schedule among patients on in-center thrice-weekly HD.

## Methods

We conducted a retrospective cohort study using data from the USRDS [12], which include the Centers for Medicare & Medicaid Services’ (CMS) inpatient and outpatient claims files and data from Consolidated Renal Operations in a Web-enabled Network (CROWNWeb) [13]. The latter is a web-based system that collects both administrative and monthly clinical data from all Medicare-certified dialysis facilities in the US. To ensure full capture of data on hospital admissions and ED visits, only patients receiving outpatient HD on a thrice-weekly schedule between January 1 and December 31, 2013, with Medicare as their primary payer, were included in this study, as administrative claims data from those with other forms of insurance and with ESRD were not available.

Using inpatient and outpatient claims, we identified five outcome events in 2013, by day-of-the-week and HD schedule. These included (i) total all-cause ED visits, (ii) total all-cause hospitalizations, (iii) ED visits not followed by a hospitalization on the same day, (iv) ED visits followed by a hospitalization on the same day (or equivalently, hospitalization preceded on the same day by an ED visit), and (v) hospitalizations not preceded on the same day by an ED visit. For each of the five outcome types, we used ICD-9-CM diagnostic codes to identify three cause-specific events: cardiovascular disease events (CVD), infections, and vascular-access complications (S1 Table). The dialysis schedule (MWF or TTS) was based on the patient’s first recorded session for each HD treatment period in 2013, based on the CROWNWeb treatment file. We excluded a small number of patients who were treated on Sundays at baseline in 2013. Race/ethnicity was categorized into five groups: Hispanic, non-Hispanic white, non-Hispanic black/African American, non-Hispanic Native American or Alaskan Native, and non-Hispanic Asian or Pacific Islander. Mean values were obtained from 2013 CROWNWeb monthly data for HD delivered treatment time, Kt/V, body mass index (BMI), and interdialytic weight gain (IDWG). Comorbidity burden was assessed from ICD-9 codes, using Deyo’s modification of Charlson’s comorbidity index, which includes 17 conditions, but we excluded kidney disease [14].

Although some HD patients would be expected to change their dialysis schedules in 2013, we did not have weekly data to do a formal time-dependent analysis with changing dialysis schedules during follow-up. Thus, a sensitivity analysis was conducted with total all-cause ED visits and hospital admissions to assess the limitation of treating dialysis schedule as a fixed baseline variable. This sensitivity analysis was therefore restricted to those patients who did not change their dialysis schedules in 2013, as determined in CROWNWeb on the day of the week that Kt/V was obtained at the end of each month.

### Statistical methods

The unadjusted incidence rate (expressed per year) of each outcome event on a given day of the week for patients on a specific dialysis schedule was estimated as the number of events occurring in that group in 2013, divided by person-years at risk [15]. Incidence rate ratios (IRR) and their corresponding 95% confidence intervals (CI), by day of the week and dialysis schedule, for all-cause outcome events were estimated by Poisson regression with robust variance estimators. These were adjusted for age, sex, race/ethnicity, the modified Charlson comorbidity index, vintage (duration on dialysis, in years), the average duration of HD sessions (in minutes), Kt/V, IDWG, and BMI. Day of the week and dialysis schedule were jointly coded as 13 indicator variables, with the MWF schedule on Sunday as the reference group. Adjusted rates for cause-specific outcomes were calculated as the expected number of events estimated by Poisson regression, divided by the number of person-years at risk.

This research was conducted as part of the USRDS Coordinating Center contract approved by the University of Michigan’s Institutional Review Board (HUM0086162). Data were analyzed using SAS version 9.4 (SAS Institute).

## Results

We identified 241,093 thrice-weekly HD patients in 2013, of whom 133,053 (55%) were on a MWF schedule and 108,040 (45%) were on a TTS schedule. Table 1 shows that patient characteristics were similar for patients on both dialysis schedules. The mean age was 62 years, 55% were men, 40% were non-Hispanic whites, and mean vintage was 4.2 years.

**Table 1:** Summary of demographic characteristics [percentage or means (standard deviation)] of in-center HD patients by dialysis schedule, in 2013.

	Monday, Wednesday, Friday	Tuesday, Thursday, Saturday
Characteristic Categories	(N = 133,053)	(N = 108,040)
Sex (Male)	55.4	55.1
Race/Ethnicity		
Hispanic	16.6	16.9
Non-Hispanic Native American	1.3	1.2
Non-Hispanic Asian	4.0	4.3
Non-Hispanic Black	37.3	38.5
Non-Hispanic White	40.7	38.9
Other/Unknown	0.1	0.1
Charlson Comorbidity Index		
0	14.9	14.9
1–3	67.0	66.9
4–6	15.1	15.1
> = 7	3.1	3.2
Time on dialysis		
Less than 6 months	8.6	9.5
6 months-3 years	36.8	38.6
More than 3 years	54.6	51.9
Primary cause of end-stage renal disease		
Diabetes	46.7	47.4
Hypertension	30.6	30.6
Glomerulonephritis	11.3	11.0
Cystic kidney disease	2.5	2.4
Other	8.8	8.6
Age (yrs.)	62.3 (14.8)	62.4 (14.7)
Years of dialysis	4.3 (3.8)	4.1 (3.7)
Delivery time (mins.)	218.9 (35.1)	220.2 (34.8)
Kt/V^a^	1.6 (0.2)	1.6 (0.2)
Interdialytic weight gain (%)	3.2 (1.7)	3.2 (1.6)
Body-Mass Index^b^	28.6 (8.0)	28.5 (8.0)

^a^ Kt/V is a unitless measure of clearance in which K is the urea clearance of the dialyzer, t is the duration of dialysis, and V is the volume of distribution of urea in the patient.

^b^ The body mass index was calculated by weight in kilograms divided by the square of the height in meters.

Among MWF patients, there were 279,602 ED visits and 164,900 hospital admissions in 2013; among those on TTS schedule, there were 235,171 ED visits and 136,774 hospitalizations. Table 2 summarizes the number and rate (and 95% CI) of all-cause outcome events of each type, by dialysis schedule and day of the week. For MWF patients, the rate of all-cause outcome events was 2.91/year for total ED visits, 1.29/year for ED visits followed by hospitalization, 1.62/year for ED visits not followed by hospitalization, 1.71/year for total hospitalizations, and 0.42/year for hospitalizations not preceded by an ED visit. All 95% CIs were very narrow due to the large numbers of outcome events. Similarly, for TTS patients, the rate of all-cause outcome events was 2.96/year for total ED visits, 1.32/year for ED visits followed by hospitalization, 1.65/year for ED visits not followed by hospitalization, 1.73/year for total hospitalization, and 0.41/year for hospitalizations not preceded by an ED visit. Crude cause-specific incidence rates (and 95% CIs) for each type of outcome are shown in S3–S7 Tables.

**Table 2:** Number and rate (per year) of all-cause total ED visits, ED visits followed by hospital admission, ED visits not followed by hospital admission, total hospital admissions, and hospital admission not preceded by an ED visit, among in-center HD patients, by dialysis schedule and day of the week.

	Monday, Wednesday, Friday		Tuesday, Thursday, Saturday	
Day	Number of Events	Rate (per year) (95% CI)	Number of Events	Rate (Per year) (95% CI)
***ED visit followed by a hospital admission***				
**Sun**	14,815	1.08 (1.06, 1.10)	11,035	0.98 (0.96, 0.99)
**Mon**	25,565	1.86 (1.84, 1.89)	18,410	1.63 (1.61, 1.65)
**Tue**	18,170	1.33 (1.31, 1.35)	18,904	1.68 (1.65, 1.70)
**Wed**	19,224	1.40 (1.38, 1.41)	14,422	1.27 (1.25, 1.29)
**Thu**	15,211	1.11 (1.09, 1.12)	15,059	1.33 (1.31, 1.35)
**Fri**	18,377	1.34 (1.32, 1.36)	13,250	1.17 (1.15, 1.19)
**Sat**	13,029	0.95 (0.93, 0.97)	13,425	1.19 (1.17, 1.21)
**Total**	124,391	1.29 (1.29, 1.30)	104,505	1.32 (1.31, 1.33)
***ED visits not followed by a hospital admission***				
**Sun**	18,814	1.37 (1.35, 1.39)	14,365	1.27 (1.25, 1.29)
**Mon**	28,995	2.11 (2.09, 2.14)	21,294	1.88 (1.86, 1.91)
**Tue**	21,063	1.54 (1.52, 1.56)	21,473	1.91 (1.88, 1.93)
**Wed**	24,169	1.75 (1.73, 1.78)	17,878	1.57 (1.55, 1.60)
**Thu**	19,691	1.43 (1.41, 1.45)	19,230	1.70 (1.67, 1.72)
**Fri**	24,347	1.77 (1.75, 1.79)	17,768	1.57 (1.54, 1.59)
**Sat**	18,132	1.32 (1.30, 1.34)	18,658	1.65 (1.63, 1.67)
**Total**	155,211	1.62 (1.61, 1.62)	130,666	1.65 (1.64, 1.65)
***Total hospital admissions***				
**Sun**	16,974	1.24 (1.22, 1.26)	12,698	1.12 (1.10, 1.14)
**Mon**	32,575	2.37 (2.35, 2.40)	24,153	2.14 (2.11, 2.16)
**Tue**	26,538	1.94 (1.92, 1.96)	25,097	2.23 (2.20, 2.26)
**Wed**	26,115	1.90 (1.87, 1.92)	20,308	1.79 (1.76, 1.81)
**Thu**	22,573	1.64 (1.62, 1.66)	20,471	1.81 (1.78, 1.83)
**Fri**	24,625	1.79 (1.77, 1.81)	18,482	1.63 (1.61, 1.65)
**Sat**	15,500	1.13 (1.11, 1.15)	15,565	1.38 (1.35, 1.40)
**Total**	164,900	1.71 (1.71, 1.72)	136,774	1.73 (1.72, 1.73)
***Hospital admission not preceded by an ED visit***				
**Sun**	2,159	0.16 (0.15, 0.16)	1,663	0.15 (0.14, 0.15)
**Mon**	7,010	0.51 (0.50, 0.52)	5,743	0.51 (0.50, 0.52)
**Tue**	8,368	0.61 (0.60, 0.63)	6,193	0.55 (0.54, 0.56)
**Wed**	6,891	0.50 (0.49, 0.51)	5,886	0.52 (0.51, 0.53)
**Thu**	7,362	0.54 (0.52, 0.55)	5,412	0.48 (0.46, 0.49)
**Fri**	6,248	0.45 (0.44, 0.47)	5,232	0.46 (0.45, 0.47)
**Sat**	2,471	0.18 (0.17, 0.19)	2,140	0.19 (0.18, 0.20)
**Total**	40,509	0.42 (0.42, 0.43)	32,269	0.41 (0.40, 0.41)

**Fig 1:**
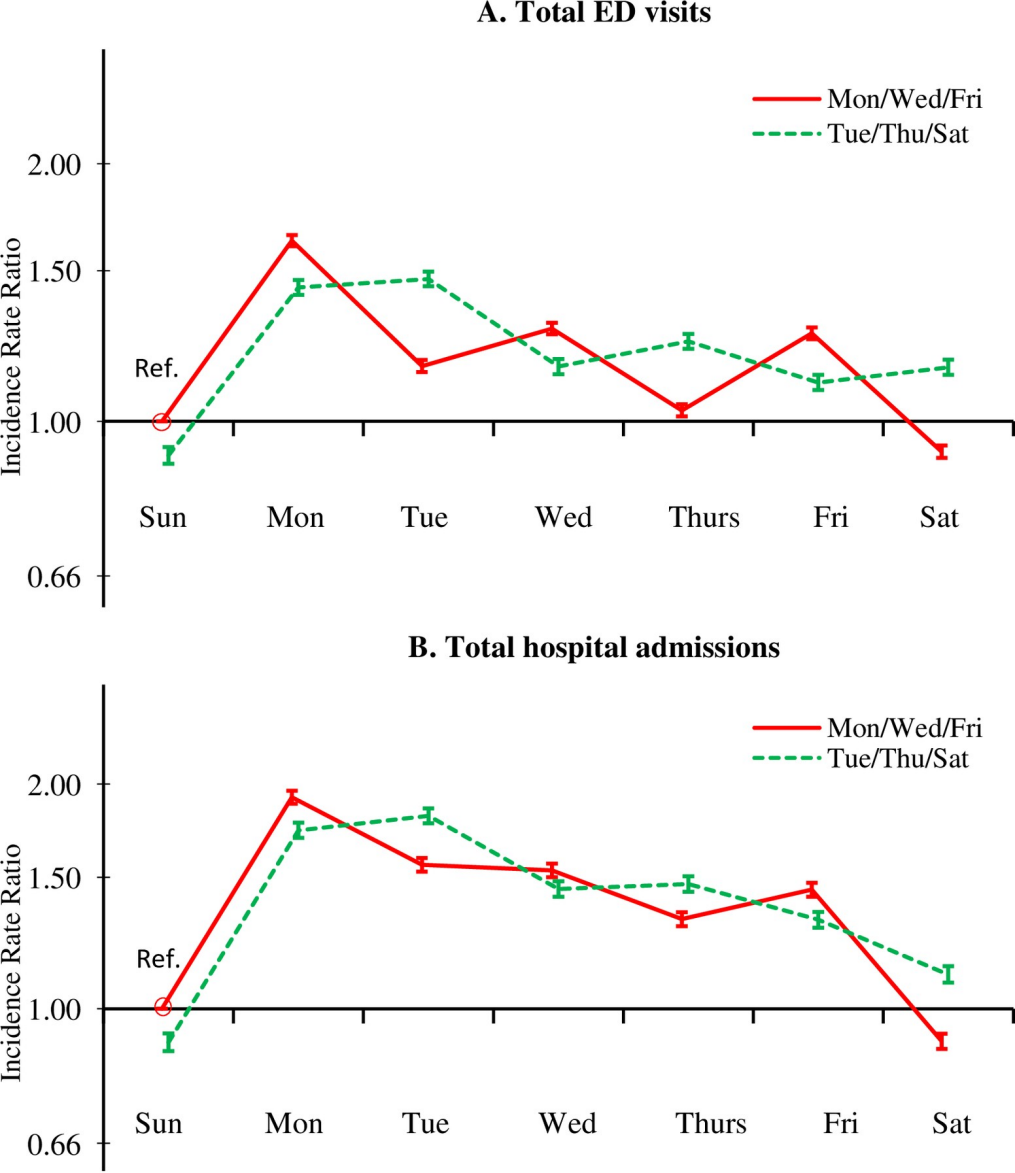
**Adjusted incidence rate ratio (95% CI) of (A) total ED visits and (B) total hospital admissions among in-center HD patients in 2013, by dialysis schedule (MWF or TTS) and day of the week, compared with the MWF group in Sunday (reference group).** Incidence rate ratios were adjusted for age, sex, race/ethnicity, the Charlson Comorbidity Index score, HD vintage, HD session length, Kt/V, IDWG, and BMI.

Fig 1 shows adjusted incidence rate ratios (IRR) of all-cause total ED visits and total hospitalizations, relative to the MWF dialysis schedule on Sunday, by dialysis schedule and day of the week. Incidence rates of both outcomes were higher on dialysis days, resulting in a visible sawtooth pattern for both outcomes. We refer to this weekly pattern as the “dialysis-day effect,” which was more pronounced for total ED visits (Fig 1A) than for total hospitalizations (Fig 1B).

We observed a lower incidence rate on weekends (Saturday and Sunday) than during the week for both outcomes (Fig 1); the rate increased appreciably between Sunday and Monday, irrespective of dialysis schedule. We refer to this pattern as the “post-weekend effect,” which in contrast to the dialysis-day effect, was stronger for total hospitalizations than for total ED visits. Thus, the two outcomes in Fig 1 reflect both patterns described so far. The incidence rates for total ED visits (Fig 1A) and hospitalizations (Fig 1B) were highest on Monday for MWF patients due to both the “post-weekend effect” and the “dialysis-day effect.”

As shown in Table 2 and Fig 1, the incidence rates for both total ED visits and hospitalizations tended to be highest at the end of the 3-day interdialytic interval, i.e., on Monday for MWF patients and on Tuesday for TTS patients. We refer to this pattern–the interaction between day of the week and dialysis schedule–as the “interdialytic-gap effect,” which was more pronounced for MWF patients. The incidence rate for both outcomes was highest on Tuesday for TTS patients due to both the “interdialytic-gap effect” and the “dialysis-day effect.”

**Fig 2:**
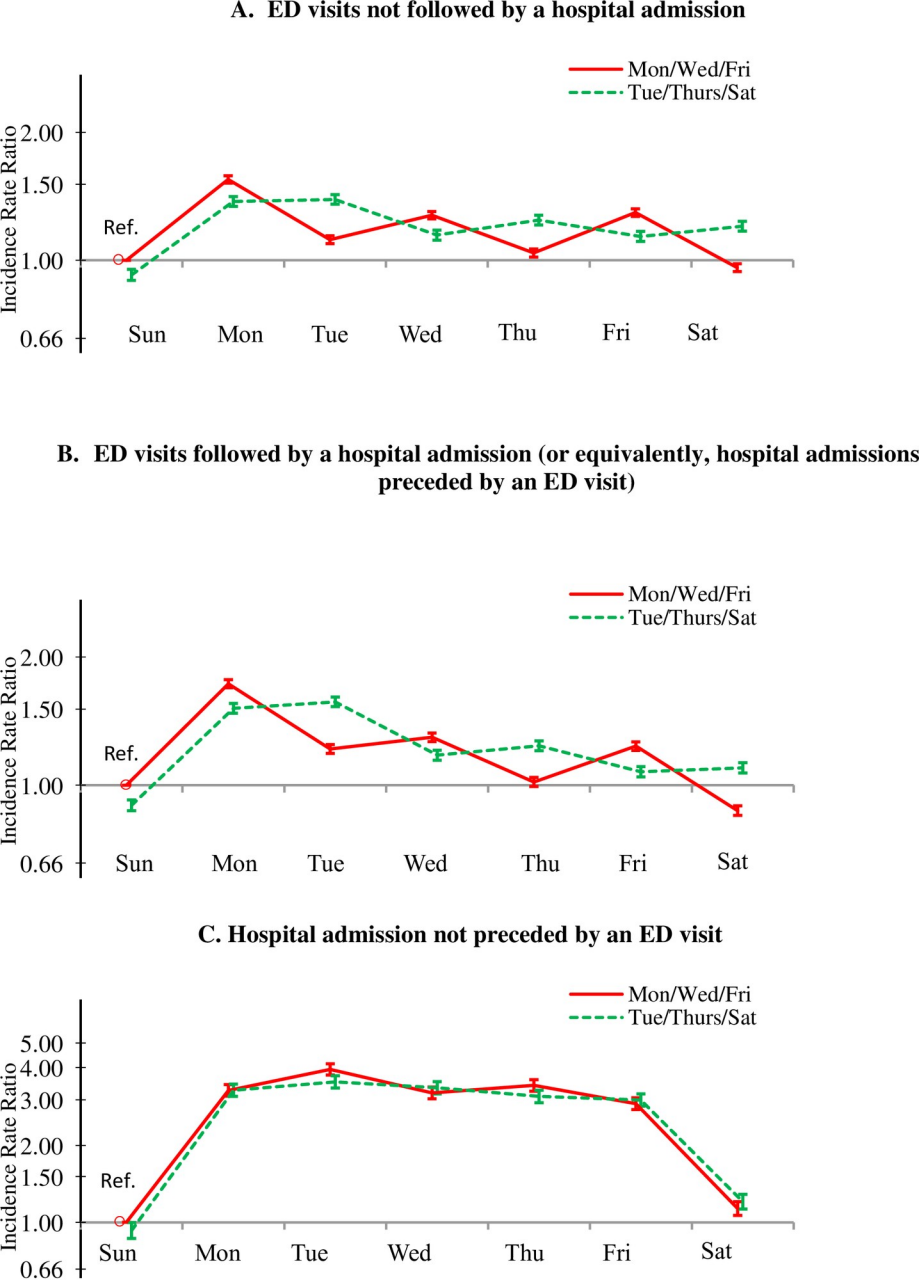
**Adjusted incidences rate ratio (95% CI) of (A) ED visits not followed by a hospital admission, (B) ED visits followed by a hospital admission, and (C) hospital admissions not preceded by an ED visit among in-center HD patients in 2013, by dialysis schedule (MWF or TTS) and day of the week, compared with the MWF group on Sunday (reference group)**. Incidence rate ratios were adjusted for age, sex, race/ethnicity, the Charlson Comorbidity Index score, HD vintage, HD session length, Kt/V, IDWG, and BMI.

Fig 2A–2C display the adjusted IRRs for the other three outcome events by day-of-the-week and dialysis schedule. The pattern of incidence rates observed for ED visits not followed by hospitalization (Fig 2A) and those followed by hospitalization (Fig 2B) were similar. Both showed the three patterns observed in Fig 1 for total ED visits: the dialysis-day effect (sawtooth pattern), the post-weekend effect (though less pronounced than in Fig 1), and the interdialytic-gap effect. In contrast, the incidence-rate pattern for hospitalization not preceded by an ED visit (Fig 2C) was distinctly different from the other outcomes. It showed a strong post-weekend effect but little dialysis-day effect or interdialytic-gap effect. Rates were nearly the same for patients in the two dialysis schedules and consistently higher Monday through Friday, without the sawtooth pattern.

**Fig 3:**
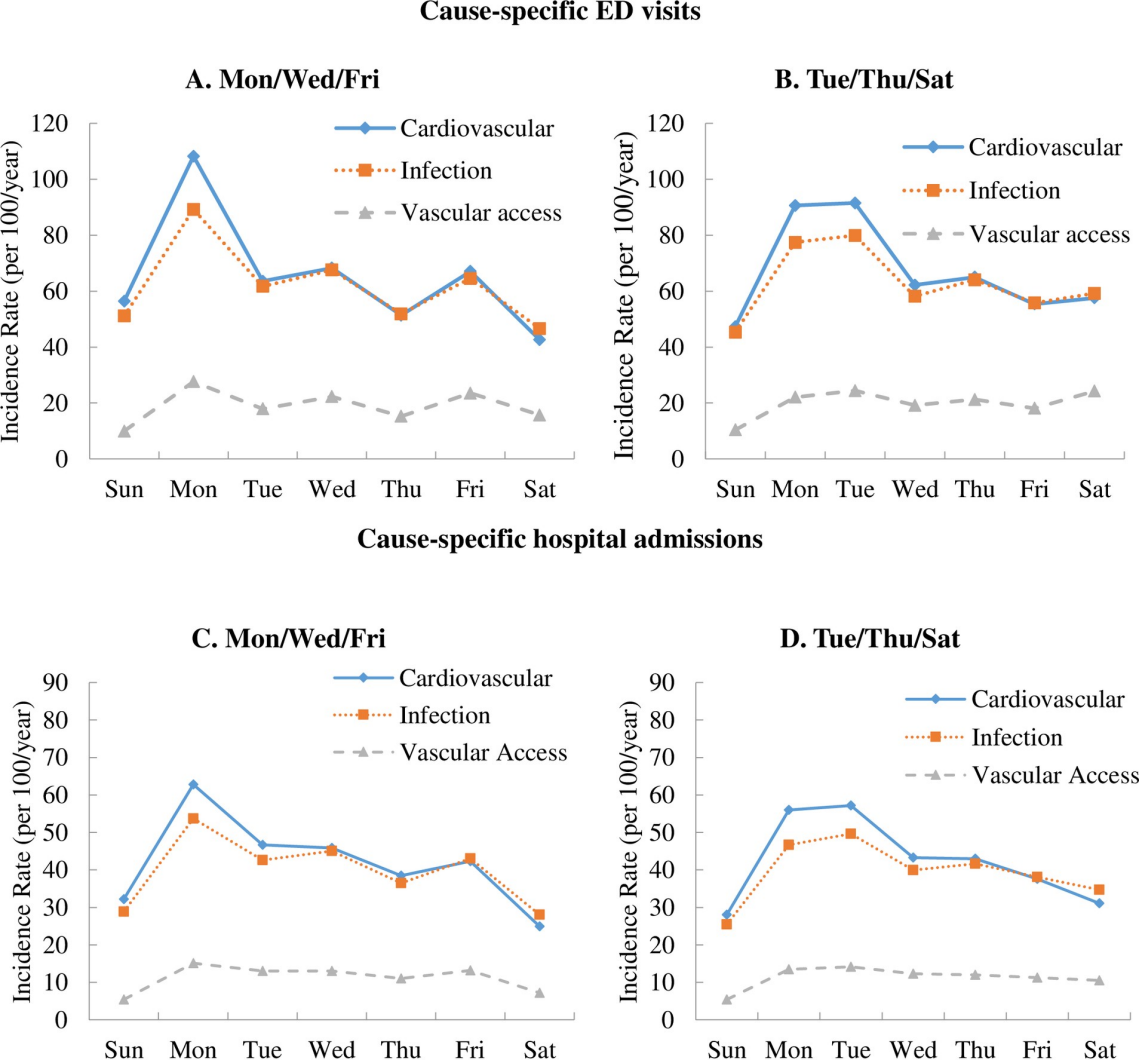
**Adjusted incidence rates (per 100/year) of (A, B) cause-specific ED visits and (C, D) cause-specific hospital admissions for in-center HD patients in 2013, by dialysis schedule (MWF or TTS), day of the week, and primary diagnosis**. Incidence rates were adjusted for age, sex, race/ethnicity, the Charlson Comorbidity Index score, HD vintage, HD session length, Kt/V, IDWG, and BMI.

Fig 3 shows the adjusted incidence rates of cause-specific ED visits (Fig 3A and 3B) and hospitalizations (Fig 3C and 3D), by dialysis schedule and day of the week. ED visits and hospitalizations for CVD and infections showed a weekly incidence pattern similar to the all-cause events in Fig 2. The rate was highest on Monday for the MWF dialysis schedule (Fig 3A and 3C) and on both Monday and Tuesday for the TTS group (Fig 3B and 3D). In contrast, the incidence rates of both ED visits and hospital admissions for vascular-access complications were lower in magnitude and showed less variation by day of the week, although with consistently lower rates on Sunday and a weak dialysis-day effect (Fig 3A). S1–S8 Figs show cause-specific and cardiovascular-related incidence rates for each type of outcome.

**Fig 4:**
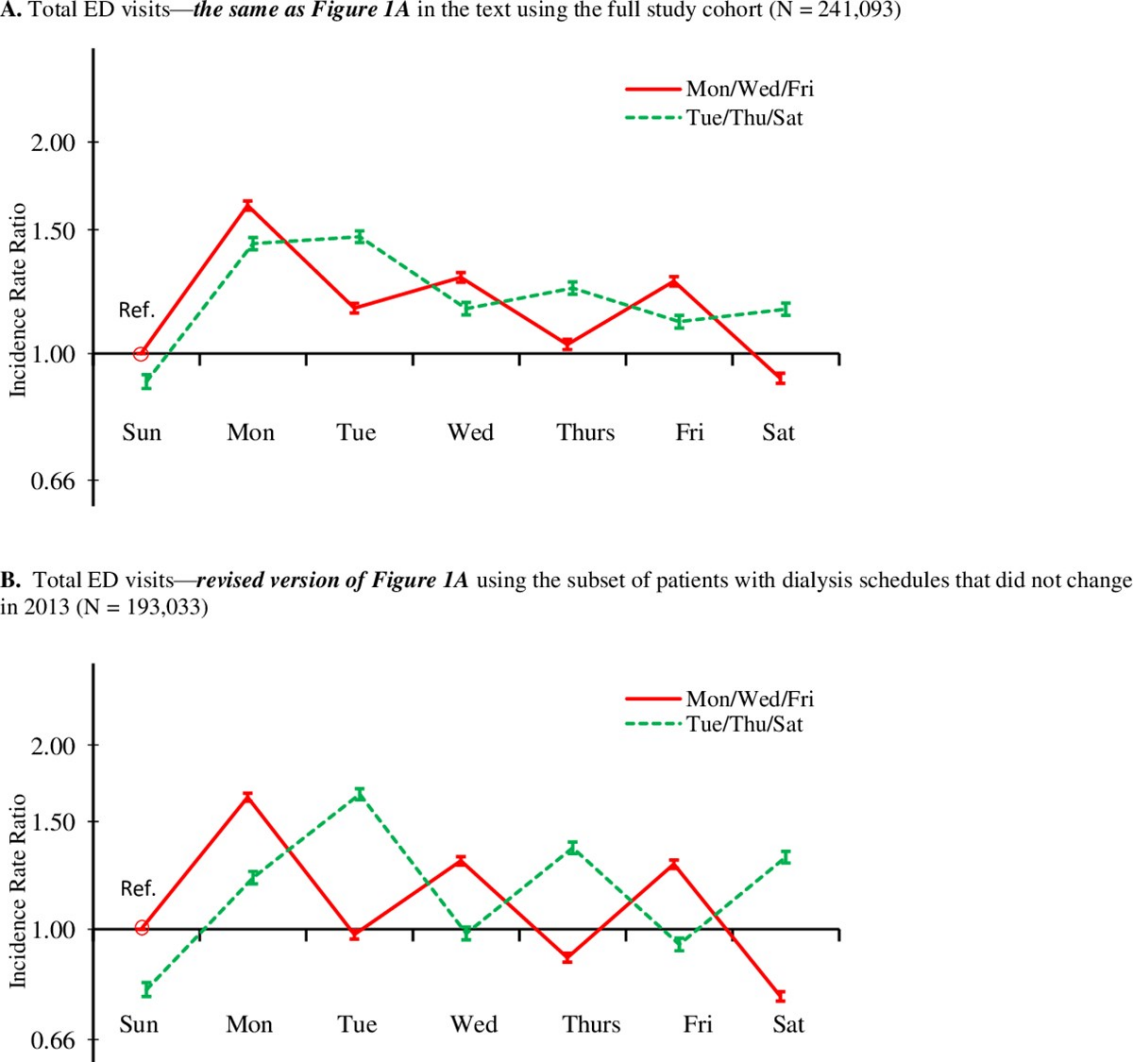
**Comparison of (A) findings from the main analysis of total ED visits in Fig 1A with (B) findings from the restricted sensitivity analysis of total ED visits**. Each figure shows the adjusted incidence rate ratio (95% CI) of total ED visits, by dialysis schedule (MWF or TTS) and day of the week, compared with the MWF group on Sunday (reference group). Incidence rate ratios were adjusted for age, sex, race/ethnicity, the Charlson Comorbidity Index score, HD vintage, HD session length, Kt/V, IDWG, and BMI.

**Fig 5:**
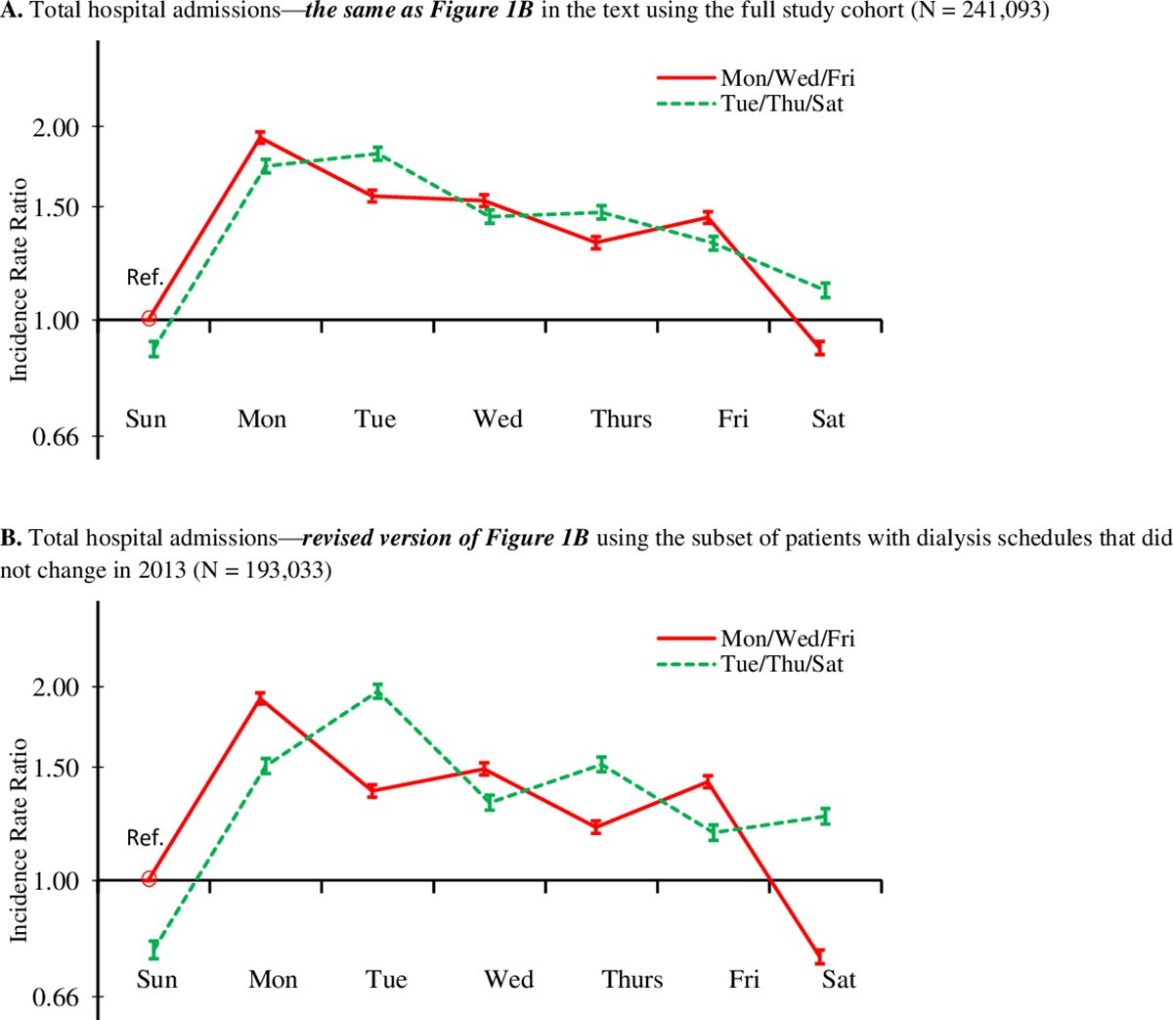
**Comparison of (A) findings from the main analysis of total hospital admissions in Fig 1B with (B) findings from the restricted sensitivity analysis of total hospital admissions.** Each figure shows the adjusted incidence rate ratio (95% CI) of total hospital admissions, by dialysis schedule (MWF or TTS) and day of the week, compared with the MWF group on Sunday (reference group). Incidence rate ratios were adjusted for age, sex, race/ethnicity, the Charlson Comorbidity Index, HD vintage, HD session length, Kt/V, IDWG, and BMI.

Results of the sensitivity analysis that was restricted to patients with the same dialysis schedule throughout 2013 are shown in Fig 4B for total ED visits and Fig 5B for total hospital admissions. For purposes of visual comparison, they are preceded by the original findings in the main analysis for total ED visits (Fig 4A) and total hospital admissions (Fig 4C). Of the 241,093 patients in the main analyses, 193,033 (80.1%) were in the restricted sample. Of the 19.9% who were excluded, 15.2% were known to have changed their dialysis schedules in 2013, and 4.7% had missing Kt/V data in one or more months so that we could not confirm the consistency of their dialysis schedules. Results of the sensitivity analyses (Figs 4B and 5B) show overall patterns that were similar to the main findings. In fact, the dialysis-day effects and interdialytic-gap effects are more pronounced in the restricted sensitivity analysis. The post-weekend effect was a less pronounced in the sensitivity analysis, especially for total ED visits, but still quite evident between Sunday and Monday for both dialysis schedules.

## Discussion

To the best of our knowledge, this is the first study to describe in detail, focusing entirely on examining the patterns of both ED visits and hospitalizations by day of the week for different outpatient dialysis schedules among thrice-weekly HD patients throughout the US. Prior studies [2–4] focused primarily on the mortality rate by day of the week and dialysis schedule and emphasized only what we have called the “interdialytic-gap effect.” By examining the outcomes of more than 500,000 ED visits and 300,000 hospitalizations in over 240,000 US HD patients and by controlling for several potential confounders, we observed three types of overlapping weekly patterns with parameters very precisely estimated (narrow 95% confidence intervals). The fact that the patterns of ED visits and hospitalizations, while somewhat similar to those previously described for mortality in the thrice-weekly hemodialysis setting, were also remarkably different from mortality pattern. This is likely due to the fact that these outcomes do not simply represent the consequences of illness *per se* but also reflect patient care-seeking behavior, access to care, and provider recommendations [16].

The dialysis-day effect, especially strong for total ED visits, is a new finding that derives from our assessment of the interaction between day of the week and dialysis schedule. While this sawtooth pattern may be the result of simply being under medical supervision on the days when HD is being delivered that produces this distinct pattern, this may be only part of the explanation. The days of dialysis sessions are often associated with both large volume and electrolyte shifts in these often frail patients with a high prevalence of comorbid illness (especially diabetes, cardiovascular disease, vascular stiffness, systolic and diastolic dysfunction, reduced heart rate variability, autonomic insufficiency, etc.) resulting in reduced circulatory and neuro-humoral adaptive responses. Consequently, there is greater potential for hemodynamic instability and high incidence of intradialytic hypotension, predisposing to complications (e.g., myocardial stunning, myocardial infarction, falls, etc.) that can result in the need for an ED visit or hospitalization. In a US study by Sands et al. [17], nearly 20% of all dialysis sessions were complicated by the occurrence of intradialytic hypotension, with appreciable facility-level variation. Intradialytic instability can predispose to both intra- and post-dialytic complications such as hypotension, cardiac arrhythmias, and post-dialysis fatigue with prolongation of recovery time post-dialysis [18]. Furthermore, patients with congestive heart failure, high interdialytic weight gain, baseline hypotension and diastolic dysfunction are especially prone to the effects of rapid ultrafiltration, itself an independent predictor of unstable dialysis sessions and mortality [10,19]. In examining cause-specific ED visit and hospitalization rates, the highest rates observed, not surprisingly, were due to congestive heart failure and dysrhythmia, predominantly early in the week for ED visits and higher on dialysis days for hospitalization (S1 and S6 Figs).

Another new finding is the very different patterns of hospitalization rates, depending on whether the hospitalization event was preceded by an ED visit or not. For admissions not preceded by an ED visit, there was a pronounced post-weekend effect (Fig 2C), but almost no dialysis-day or interdialytic-gap effects. The likely explanation is that admissions without a preceding ED visit were elective or semi-elective in nature, thereby occurring at a constant higher rate during the week, but not over the weekend.

We also found that the all-cause hospital admission and ED visit rates were relatively high on Mondays in both dialysis schedules, a pattern not reported in previous mortality studies (S1 and S7 Figs). This post-weekend effect likely results from deferring Saturday and Sunday ED visits and hospitalization until Monday, regardless of dialysis schedule. Findings from two studies [4,5] of HD patients in the United Kingdom and Japan did not show a consistent post-weekend effect, suggesting that this pattern may not apply in other countries.

Our findings of high hospitalization and ED visit rates early in the week and on dialysis days, with relatively low occurrences during the weekend, have important implications for patients, clinicians, and policymakers. This is especially true for the large number of patients on thrice-weekly HD. For example, it may indicate that patients are either reluctant to seek help and defer their ED visit or in some instances, feel discouraged from seeking medical attention over the weekend. Other investigators have studied whether higher cause-specific mortality rates occur on the weekend [20–22]. Sakhuja et al. [23] reported that maintenance dialysis patients admitted on weekends had higher all-cause mortality rates during their hospital stays and for three days after discharge than did patients admitted on weekdays. These findings suggest that those who are hospitalized over the weekend are likely seriously ill and cannot defer their visit to the ED or hospital to early the following week, and may risk succumbing to their illness.

One limitation of our study is the lack information on the exact time of occurrence of morbid events in relation to the scheduled (or possibly missed or shortened) dialysis sessions on the day of dialysis. Consequently we could not determine whether those outcome events occurred before, during, after or instead of the dialysis session. Thus, we cannot make inferences about what caused the excess ED visits or hospitalizations on those days when HD sessions were scheduled. It may simply be that caregivers provide referrals to EDs or hospitals during HD sessions, not that patients are adversely affected by their treatment. Similarly, to explain the “interdialytic-gap effect,” we cannot determine whether the long interval since the last HD treatment exacerbated the patient’s condition or if the patient simply waited longer to seek medical attention on the next dialysis day. We did not have access to data from the dialysis session or electronic health records, such as blood pressure or other vital signs, to study phenomena such as intradialytic instability, intradialytic hypotension, patient symptoms, or treatments received during the HD session. We were, therefore, unable to link the higher rate of ED visits and admissions on dialysis days to any potential intradialytic complications per se; however, this possibility cannot be ruled out and should lend itself to future investigation.

As noted in the Methods, our main analyses were based on treating dialysis schedule as a fixed baseline variable. Because we did not have sufficient data to do a time-dependent analysis, we conducted a sensitivity analysis of our two primary outcomes, total all-cause ED visits and hospitalizations, which was restricted to HD patients whose dialysis schedules did not change in 2013. We found that the dialysis-day and interdialytic-gap effects were more pronounced in the restricted sample, suggesting that these patterns were actually stronger than they appeared in the main analysis when treating dialysis schedule as a fixed baseline variable.

Another limitation in this observational study is possible residual (uncontrolled) confounding due to unmeasured risk factors for hospital visits that are associated with day of the week and/or dialysis schedule (e.g., patient nonadherence with dialysis schedule, socioeconomic status, work schedule, or physician availability). We did find, however, that patients on different dialysis schedules were similar on a variety of patient characteristics (Table 1), and we adjusted for several covariates using Poisson regression. Nevertheless, there may have been other unmeasured confounders, and adjustment for time-varying covariates treated as fixed at baseline may have been compromised by the fact that they were not measured shortly before outcome events were observed in patients.

Finally, this was an exploratory study in which our objective was to describe weekly patterns in the occurrence of ED visits and hospitalizations among hemodialysis patients. The three patterns we described–dialysis-day, post-weekend, and interdialytic-gap “effects”–are not independent of each other, as previously noted; and we did not infer them to be effects in the causal sense. Our aim was not causal inference but to promote the generation of new hypotheses and research on the use of hospital services in this patient population and to assist policy makers, administrators and clinicians who must plan for the care of these patients.

In conclusion, we conducted a comprehensive analysis of hospitalizations and ED visits among HD patients in the entire US in 2013, and found that the incidence rates of these outcomes varied systematically by dialysis schedule, day of the week, and type of outcome. The underlying mechanisms for the weekly patterns we observed remain speculative but may well relate to the “unphysiology” of thrice-weekly dialysis [24], and/or the widely prevalent practice of relatively short HD sessions and rapid ultrafiltration rates that contribute to the high incidence of intradialytic hypotension or hemodynamic instability in the hemodialysis population.

Despite its limitations, our study was national in scope with very precise estimation of outcome rates and rate ratios, and it helps to extend our understanding of healthcare resource utilization among thrice-weekly HD patients in the US. We cannot say, however, the extent to which our findings may be generalizable to HD populations in other countries. Nevertheless, the study by Fotheringham et al. [25], which was conducted in 7 European countries, yielded findings for all-cause hospitalization similar to ours: All three weekly patterns that we described are evident in their results (Fig 1B), though the authors focused attention on what we call the interdialytic-gap effect.

Our study should serve to stimulate further research into the safety of dialysis sessions as currently practiced. Additionally, there should be a serious reexamination of factors underlying both high mortality and morbidity after the weekend or long gap in dialysis. In particular, if the dialysis-day excess in ED visits and hospitalizations could be reduced in this high-risk patient population, it would go a long way to help improve both patient experience and health outcomes while reducing cost of care for dialysis patients in this country.

## Supporting information

S1 Table Definitions of causes and types of admission, and their principal ICD-9-CM diagnosis codes. (DOCX)

S2 Table All-cause total ED visits, ED visits followed by a hospital admission, ED visits not followed by a hospital admission, total hospital admission, and hospital admission not preceded by an ED visits incidence rate ratios (IRR) among in-center HD patients, by dialysis schedule (MWF or TTS) and day of the week, with Sunday Mon/Wed/Fri group as reference. (DOCX)

S3 Table All-cause and cause-specific total ED visits rate* (per year) among in-center HD patients, by dialysis schedule (MWF or TTS), day of the week, and primary cause of admission. (DOCX)

S4 Table All-cause and cause-specific ED visits followed by a hospital admission rate* (per year) among in-center HD patients, by dialysis schedule (MWF or TTS), day of the week, and primary cause of admission. (DOCX)

S5 Table All-cause and cause-specific ED visits not followed by a hospital admission rate* (per year) among in-center HD patients, by dialysis schedule (MWF or TTS), day of the week, and primary cause of admission. (DOCX)

S6 Table All-cause and cause-specific total hospital admission rate* (per year) among in-center HD patients, by dialysis schedule (MWF or TTS), day of the week, and primary cause of admission. (DOCX)

S7 Table All-cause and cause-specific hospital admission not preceded by an ED visit rate* (per year) among in-center HD patients, by dialysis schedule (MWF or TTS), day of the week, and primary cause of admission. (DOCX)

S1 Fig Adjusted incidence rate (per 100/year)* of cardiovascular related total ED visits for in-center HD patients in 2013, by dialysis schedule ((a) MWF vs. (b) TTS), day of the week and types of cardiovascular diseases.
(DOCX)

S2 Fig Adjusted incidence rate (per 100/year)* of cause-specific ED visits followed by a hospital admission for in-center HD patients in 2013, by dialysis schedule ((a) MWF vs. (b) TTS), day of the week and primary diagnosis of ED visits.
(DOCX)

S3 Fig Adjusted incidence rate (per 100/year)* of cardiovascular related ED visits followed by a hospital admission for in-center HD patients in 2013, by dialysis schedule ((a) MWF vs. (b) TTS), day of the week and types of cardiovascular diseases.
(DOCX)

S4 Fig Adjusted incidence rate (per 100/year)* of cause-specific ED visits not followed by a hospital admission for in-center HD patients in 2013, by dialysis schedule ((a) MWF vs. (b) TTS), day of the week and primary diagnosis of ED visits.
(DOCX)

S5 Fig Adjusted incidence rate (per 100/year)* of cardiovascular related ED visits not followed by a hospital admission for in-center HD patients in 2013, by dialysis schedule ((a) MWF vs. (b) TTS), day of the week and types of cardiovascular diseases.
(DOCX)

S6 Fig Adjusted incidence rate (per 100/year)* of total cardiovascular-admission for in-center HD patients in 2013, by dialysis schedule ((a) MWF vs. (b) TTS) and types of cardiovascular diseases.
(DOCX)

S7 Fig Adjusted incidence rate (per 100/year)* of cause-specific hospital admission not preceded by an ED visit for in-center HD patients in 2013, by dialysis schedule ((a) MWF vs. (b) TTS), day of the week and primary diagnosis of admission.
(DOCX)

S8 Fig Adjusted incidence rate (per 100/year)* of cardiovascular related hospital admission not preceded by an ED visit for in-center HD patients in 2013, by dialysis schedule ((a) MWF vs. (b) TTS), day of the week and types of cardiovascular diseases.
(DOCX)
