# Supplementary material for: Emergency department visits and hospitalizations among hemodialysis patients by day of the week and dialysis schedule in the United States
Source: PLoS One. 2019 Aug 15;14(8):e0220966. doi: 10.1371/journal.pone.0220966 (PMC6695146; doi:10.1371/journal.pone.0220966)
Supplement: S1 Table — (DOCX) [file pone.0220966.s001.docx]

## S1 Table. Definitions of causes and types of admission, and their principal ICD-9-CM diagnosis codes

| \| **Cause/Type** \| \| --- \| | \| **Principal ICD-9-CM Diagnosis Codes** \| \| --- \| |
| --- | --- | --- | --- |
| **Cardiovascular** | 276.6, 394–398.99, 401–405, 410–420, 421.9, 422.90, 422.99, 423–438, and 440–459 |
| **Acute myocardial infarction** | 410 |
| **Congestive heart failure (CHF)** | 428 |
| **Stroke** | 430-434 |
| **Dysrhythmia** | 426-427 |
| **Infection** | 001–139, 254.1, 320–326, 331.81, 372–372.39, 373.0–373.2, 382–382.4, 383, 386.33, 386.35, 388.60, 390–393, 421–421.1, 422.0, 422.91–422.93, 460–466, 472–474.0, 475–476.1, 478.21–478.24, 478.29, 480–490, 491.1, 494, 510–511, 513.0, 518.6, 519.01, 522.5, 522.7, 527.3, 528.3, 540–542, 566–567.9, 569.5, 572–572.1, 573.1–573.3, 575–575.12, 590–590.9, 595–595.4, 597–597.89, 598.0, 599.0, 601–601.9, 604–604.9, 607.1, 607.2, 608.0, 608.4, 611.0, 614–616.1, 670, 680–686.9, 706.0, 711–711.9, 790.7–790.8, 996.60–996.69, 997.62, 998.5, and 999.3 |
| **Vascular access** | 996.1, 996.62, 999.31, 996.73 and V561 |
