# Supplementary material for: Emergency department visits and hospitalizations among hemodialysis patients by day of the week and dialysis schedule in the United States
Source: PLoS One. 2019 Aug 15;14(8):e0220966. doi: 10.1371/journal.pone.0220966 (PMC6695146; doi:10.1371/journal.pone.0220966)
Supplement: S2 Table — (DOCX) [file pone.0220966.s002.docx]

## S2 Table. All-cause total ED visits, ED visits followed by a hospital admission, ED visits not followed by a hospital admission, total hospital admission, and hospital admission not preceded by an ED visits incidence rate ratios (IRR) among in-center HD patients, by dialysis schedule (MWF or TTS) and day of the week, with Sunday Mon/Wed/Fri group as reference

| **Day** | **MWF** | **TTS** |
| --- | --- | --- |
| ***Total ED visits*** | | |
| **Sun** | 1.00 (1.00, 1.00) | 0.92 (0.90, 0.94) |
| **Mon** | 1.63 (1.60, 1.65) | 1.44 (1.41, 1.47) |
| **Tue** | 1.17 (1.15, 1.19) | 1.46 (1.44, 1.49) |
| **Wed** | 1.28 (1.26, 1.30) | 1.17 (1.14, 1.19) |
| **Thu** | 1.03 (1.02, 1.05) | 1.24 (1.22, 1.27) |
| **Fri** | 1.27 (1.25, 1.29) | 1.12 (1.09, 1.14) |
| **Sat** | 0.92 (0.91, 0.94) | 1.16 (1.14, 1.18) |
| ***ED visit followed by a hospital admission*** | | |
| **Sun** | 1.00 (1.00, 1.00) | 0.90 (0.87, 0.92) |
| **Mon** | 1.73 (1.69, 1.76) | 1.51 (1.47, 1.55) |
| **Tue** | 1.22 (1.19, 1.24) | 1.57 (1.53, 1.61) |
| **Wed** | 1.29 (1.26, 1.32) | 1.18 (1.14, 1.21) |
| **Thu** | 1.02 (0.99, 1.04) | 1.24 (1.20, 1.27) |
| **Fri** | 1.23 (1.21, 1.26) | 1.08 (1.05, 1.11) |
| **Sat** | 0.87 (0.85, 0.89) | 1.10 (1.07, 1.13) |
| ***ED visits not followed by a hospital admission*** | | |
| **Sun** | 1.00 (1.00, 1.00) | 0.92 (0.90, 0.95) |
| **Mon** | 1.55 (1.52, 1.58) | 1.37 (1.34, 1.41) |
| **Tue** | 1.12 (1.09, 1.14) | 1.39 (1.35, 1.42) |
| **Wed** | 1.28 (1.25, 1.30) | 1.15 (1.11, 1.18) |
| **Thu** | 1.04 (1.02, 1.06) | 1.24 (1.21, 1.27) |
| **Fri** | 1.29 (1.27, 1.32) | 1.14 (1.11, 1.17) |
| **Sat** | 0.96 (0.94, 0.98) | 1.20 (1.17, 1.23) |
| ***Total hospital admissions*** | | |
| **Sun** | 1.00 (1.00, 1.00) | 0.91 (0.89, 0.94) |
| **Mon** | 1.93 (1.89, 1.97) | 1.74 (1.70, 1.79) |
| **Tue** | 1.57 (1.54, 1.60) | 1.81 (1.77, 1.86) |
| **Wed** | 1.54 (1.50, 1.57) | 1.46 (1.42, 1.49) |
| **Thu** | 1.33 (1.30, 1.36) | 1.47 (1.44, 1.51) |
| **Fri** | 1.45 (1.42, 1.48) | 1.33 (1.29, 1.36) |
| **Sat** | 0.91 (0.89, 0.93) | 1.11 (1.09, 1.14) |
| ***Hospital admission not preceded by an ED visit*** | | |
| **Sun** | 1.00 (1.00, 1.00) | 0.93 (0.87, 1.00) |
| **Mon** | 3.27 (3.10, 3.44) | 3.28 (3.10, 3.46) |
| **Tue** | 3.95 (3.75, 4.15) | 3.53 (3.34, 3.73) |
| **Wed** | 3.20 (3.03, 3.37) | 3.34 (3.16, 3.53) |
| **Thu** | 3.42 (3.24, 3.60) | 3.10 (2.93, 3.27) |
| **Fri** | 2.90 (2.75, 3.06) | 3.00 (2.83, 3.17) |
| **Sat** | 1.13 (1.06, 1.20) | 1.20 (1.13, 1.28) |
