# Supplementary material for: Emergency department visits and hospitalizations among hemodialysis patients by day of the week and dialysis schedule in the United States
Source: PLoS One. 2019 Aug 15;14(8):e0220966. doi: 10.1371/journal.pone.0220966 (PMC6695146; doi:10.1371/journal.pone.0220966)
Supplement: S3 Table — (DOCX) [file pone.0220966.s003.docx]

## S3 Table. All-cause and cause-specific total ED visits rate* (per year) among in-center HD patients, by dialysis schedule (MWF or TTS), day of the week, and primary cause of admission

|  | MWF | |  | TTS | |
| --- | --- | --- | --- | --- | --- |
| Day | **Number of Events** | **Rate**  **(95% CI, per year)** |  | **Number of Events** | **Rate**  **(95% CI, per year)** |
| *Total ED visits* | | | | | |
| Sun | 33,629 | 2.45 (2.42,2.48) |  | 25,400 | 2.25 (2.22,2.27) |
| Mon | 54,560 | 3.98 (3.94,4.01) |  | 39,704 | 3.51 (3.48,3.55) |
| Tue | 39,233 | 2.87 (2.84,2.89) |  | 40,377 | 3.58 (3.56,3.63) |
| Wed | 43,393 | 3.15 (3.12,3.18) |  | 32,300 | 2.84 (2.81,2.88) |
| Thu | 34,902 | 2.54 (2.51,2.56) |  | 34,289 | 3.03 (2.99,3.06) |
| Fri | 42,724 | 3.11 (3.08,3.13) |  | 31,018 | 2.73 (2.70,2.76) |
| Sat | 31,161 | 2.27 (2.24,2.29) |  | 32,083 | 2.84 (2.80,2.87) |
| *Cardiovascular-related ED visits* | | | | | |
| Sun | 5,915 | 0.43 (0.42,0.44) |  | 4,166 | 0.37 (0.36,0.38) |
| Mon | 10,530 | 0.77 (0.75,0.78) |  | 7,434 | 0.66 (0.64,0.67) |
| Tue | 6,727 | 0.49 (0.48,0.50) |  | 7,545 | 0.67 (0.65,0.68) |
| Wed | 7,588 | 0.55 (0.54,0.56) |  | 5,615 | 0.49 (0.48,0.51) |
| Thu | 5,829 | 0.42 (0.41,0.43) |  | 5,840 | 0.52 (0.50,0.53) |
| Fri | 7,340 | 0.53 (0.52,0.55) |  | 5,101 | 0.45 (0.44,0.46) |
| Sat | 4,780 | 0.35 (0.34,0.36) |  | 5,043 | 0.45 (0.43,0.46) |
| *Infection-related ED visits* | | | | | |
| Sun | 5,999 | 0.44 (0.43,0.45) |  | 4,383 | 0.39 (0.38,0.40) |
| Mon | 9,163 | 0.67 (0.65,0.68) |  | 6,711 | 0.59 (0.58,0.61) |
| Tue | 6,813 | 0.50 (0.49,0.51) |  | 6,731 | 0.60 (0.58,0.61) |
| Wed | 7,567 | 0.55 (0.54,0.56) |  | 5,482 | 0.48 (0.47,0.50) |
| Thu | 5,948 | 0.43 (0.42,0.44) |  | 5,822 | 0.51 (0.50,0.53) |
| Fri | 7,328 | 0.53 (0.52,0.54) |  | 5,358 | 0.47 (0.46,0.48) |
| Sat | 5,528 | 0.40 (0.39,0.41) |  | 5,552 | 0.49 (0.48,0.50) |
| *Vascular access-related visits* | | | | | |
| Sun | 1,206 | 0.09 (0.08,0.09) |  | 1,057 | 0.09 (0.09,0.10) |
| Mon | 3,119 | 0.23 (0.22,0.24) |  | 2,000 | 0.18 (0.17,0.18) |
| Tue | 2,103 | 0.15 (0.15,0.16) |  | 2,276 | 0.20 (0.19,0.21) |
| Wed | 2,682 | 0.19 (0.19,0.20) |  | 1,888 | 0.17 (0.16,0.17) |
| Thu | 1,851 | 0.13 (0.13,0.14) |  | 2,051 | 0.18 (0.17,0.19) |
| Fri | 2,871 | 0.21 (0.20,0.22) |  | 1,796 | 0.16 (0.15,0.17) |
| Sat | 1,939 | 0.14 (0.13,0.15) |  | 2,420 | 0.21 (0.21,0.22) |

*****Each rate was computed as the number of hospital admissions during follow-up in a group, divided by the amount of person-*years* at risk of hospitalization in that group; thus, the unit of each rate is ‘per *year*.’
