# Supplementary material for: Emergency department visits and hospitalizations among hemodialysis patients by day of the week and dialysis schedule in the United States
Source: PLoS One. 2019 Aug 15;14(8):e0220966. doi: 10.1371/journal.pone.0220966 (PMC6695146; doi:10.1371/journal.pone.0220966)
Supplement: S4 Table — (DOCX) [file pone.0220966.s004.docx]

## S4 Table. All-cause and cause-specific ED visits followed by a hospital admission rate* (per year) among in-center HD patients, by dialysis schedule (MWF or TTS), day of the week, and primary cause of admission

|  | MWF | |  | TTS | |
| --- | --- | --- | --- | --- | --- |
| Day | **Number of Events** | **Rate**  **(95% CI, per year)** |  | **Number of Events** | **Rate**  **(95% CI, per year)** |
| *All-cause ED visits followed by a hospital admission* | | | | | |
| Sun | 14,815 | 1.08 (1.06,1.10) |  | 11,035 | 0.98 (0.96,0.99) |
| Mon | 25,565 | 1.86 (1.84,1.89) |  | 18,410 | 1.63 (1.61,1.65) |
| Tue | 18,170 | 1.33 (1.31,1.35) |  | 18,904 | 1.68 (1.65,1.70) |
| Wed | 19,224 | 1.40 (1.38,1.41) |  | 14,422 | 1.27 (1.25,1.29) |
| Thu | 15,211 | 1.11 (1.09,1.12) |  | 15,059 | 1.33 (1.31,1.35) |
| Fri | 18,377 | 1.34 (1.32,1.36) |  | 13,250 | 1.17 (1.15,1.19) |
| Sat | 13,029 | 0.95 (0.93,0.97) |  | 13,425 | 1.19 (1.17,1.21) |
| *Cardiovascular-related ED visits followed by a hospital admission* | | | | | |
| Sun | 4,015 | 0.29 (0.28,0.30) |  | 2,832 | 0.25 (0.24,0.26) |
| Mon | 6,944 | 0.51 (0.49,0.52) |  | 5,044 | 0.45 (0.43,0.46) |
| Tue | 4,508 | 0.33 (0.32,0.34) |  | 5,062 | 0.45 (0.44,0.45) |
| Wed | 4,826 | 0.35 (0.34,0.36) |  | 3,631 | 0.32 (0.31,0.33) |
| Thu | 3,703 | 0.27 (0.26,0.28) |  | 3,669 | 0.32 (0.31,0.33) |
| Fri | 4,598 | 0.33 (0.32,0.34) |  | 3,215 | 0.28 (0.27,0.29) |
| Sat | 3,056 | 0.22 (0.21,0.23) |  | 3,163 | 0.28 (0.27,0.29) |
| *Infection-related ED visits followed by a hospital admission* | | | | | |
| Sun | 3,694 | 0.27 (0.26,0.28) |  | 2,699 | 0.24 (0.23,0.25) |
| Mon | 6,341 | 0.46 (0.45,0.47) |  | 4,488 | 0.40 (0.39,0.41) |
| Tue | 4,593 | 0.34 (0.33,0.35) |  | 4,733 | 0.42 (0.41,0.43) |
| Wed | 5,148 | 0.37 (0.36,0.38) |  | 3,681 | 0.32 (0.31,0.33) |
| Thu | 3,907 | 0.28 (0.28,0.29) |  | 4,005 | 0.35 (0.34,0.36) |
| Fri | 4,957 | 0.36 (0.35,0.37) |  | 3,504 | 0.31 (0.30,0.32) |
| Sat | 3,513 | 0.26 (0.25,0.26) |  | 3,664 | 0.32 (0.31,0.33) |
| *Vascular-access-related ED visits followed by a hospital admission* | | | | | |
| Sun | 658 | 0.05 (0.04,0.05) |  | 546 | 0.05 (0.05,0.05) |
| Mon | 1,452 | 0.11 (0.10,0.11) |  | 1,000 | 0.09 (0.09,0.10) |
| Tue | 1,031 | 0.08 (0.07,0.08) |  | 1,022 | 0.09 (0.09,0.10) |
| Wed | 1,180 | 0.09 (0.08,0.09) |  | 867 | 0.08 (0.07,0.08) |
| Thu | 820 | 0.06 (0.06,0.06) |  | 887 | 0.08 (0.08,0.09) |
| Fri | 1,226 | 0.09 (0.09,0.10) |  | 811 | 0.07 (0.07,0.08) |
| Sat | 840 | 0.06 (0.06,0.07) |  | 1,017 | 0.09 (0.09,0.10) |

*Each rate was computed as the number of hospital admissions during follow-up in a group, divided by the amount of person-*years* at risk of hospitalization in that group; thus, the unit of each rate is ‘per *year*.’
