# Supplementary material for: Emergency department visits and hospitalizations among hemodialysis patients by day of the week and dialysis schedule in the United States
Source: PLoS One. 2019 Aug 15;14(8):e0220966. doi: 10.1371/journal.pone.0220966 (PMC6695146; doi:10.1371/journal.pone.0220966)
Supplement: S5 Table — (DOCX) [file pone.0220966.s005.docx]

## S5 Table. All-cause and cause-specific ED visits not followed by a hospital admission rate* (per year) among in-center HD patients, by dialysis schedule (MWF or TTS), day of the week, and primary cause of admission

|  | MWF | |  | TTS | |
| --- | --- | --- | --- | --- | --- |
| Day | Number of Events | Rate (95% CI)  (per year) |  | Number of Events | Rate (95% CI)  (per year) |
| *All-cause ED visits not followed by a hospital admission* | | | | | |
| Sun | 18,814 | 1.37 (1.35,1.39) |  | 14,365 | 1.27 (1.25,1.29) |
| Mon | 28,995 | 2.11 (2.09,2.14) |  | 21,294 | 1.88 (1.86,1.91) |
| Tue | 21,063 | 1.54 (1.52,1.56) |  | 21,473 | 1.91 (1.88,1.93) |
| Wed | 24,169 | 1.75 (1.73,1.78) |  | 17,878 | 1.57 (1.55,1.60) |
| Thu | 19,691 | 1.43 (1.41,1.45) |  | 19,230 | 1.70 (1.67,1.72) |
| Fri | 24,347 | 1.77 (1.75,1.79) |  | 17,768 | 1.57 (1.54,1.59) |
| Sat | 18,132 | 1.32 (1.30,1.34) |  | 18,658 | 1.65 (1.63,1.67) |
| *Cardiovascular-related ED visits* | | | | | |
| Sun | 1,900 | 0.14 (0.13,0.14) |  | 1,334 | 0.12 (0.11,0.12) |
| Mon | 3,586 | 0.26 (0.25,0.27) |  | 2,390 | 0.21 (0.20,0.22) |
| Tue | 2,219 | 0.16 (0.16,0.17) |  | 2,483 | 0.22 (0.21,0.23) |
| Wed | 2,762 | 0.20 (0.19,0.21) |  | 1,984 | 0.17 (0.17,0.18) |
| Thu | 2,126 | 0.15 (0.15,0.16) |  | 2,171 | 0.19 (0.18,0.20) |
| Fri | 2,742 | 0.20 (0.19,0.21) |  | 1,886 | 0.17 (0.16,0.17) |
| Sat | 1,724 | 0.13 (0.12,0.13) |  | 1,880 | 0.17 (0.16,0.17) |
| *Infection-related ED visits* | | | | | |
| Sun | 2,299 | 0.16 (0.16,0.17) |  | 1,589 | 0.14 (0.13,0.15) |
| Mon | 2,711 | 0.20 (0.19,0.21) |  | 2,104 | 0.19 (0.18,0.19) |
| Tue | 2,092 | 0.15 (0.15,0.16) |  | 1,883 | 0.17 (0.16,0.17) |
| Wed | 2,314 | 0.17 (0.16,0.17) |  | 1,684 | 0.15 (0.14,0.16) |
| Thu | 1,947 | 0.14 (0.14,0.15) |  | 1,733 | 0.15 (0.15,0.16) |
| Fri | 2,288 | 0.17 (0.16,0.17) |  | 1,760 | 0.16 (0.15,0.16) |
| Sat | 1,929 | 0.14 (0.13,0.15) |  | 1,818 | 0.16 (0.15,0.17) |
| *Vascular access-related visits* | | | | | |
| Sun | 531 | 0.04 (0.04,0.04) |  | 493 | 0.04 (0.04,0.05) |
| Mon | 1,643 | 0.12 (0.11,0.13) |  | 974 | 0.09 (0.08,0.09) |
| Tue | 1,041 | 0.08 (0.07,0.08) |  | 1,231 | 0.11 (0.10,0.12) |
| Wed | 1,478 | 0.11 (0.10,0.11) |  | 990 | 0.09 (0.08,0.09) |
| Thu | 1,006 | 0.07 (0.07,0.08) |  | 1,139 | 0.10 (0.09,0.11) |
| Fri | 1,626 | 0.12 (0.11,0.12) |  | 965 | 0.09 (0.08,0.09) |
| Sat | 1,064 | 0.08 (0.07,0.08) |  | 1,377 | 0.12 (0.12,0.13) |

*Each rate was computed as the number of hospital admissions during follow-up in a group, divided by the amount of person-*years* at risk of hospitalization in that group; thus, the unit of each rate is ‘per *year*.’
