# Supplementary material for: Emergency department visits and hospitalizations among hemodialysis patients by day of the week and dialysis schedule in the United States
Source: PLoS One. 2019 Aug 15;14(8):e0220966. doi: 10.1371/journal.pone.0220966 (PMC6695146; doi:10.1371/journal.pone.0220966)
Supplement: S6 Table — (DOCX) [file pone.0220966.s006.docx]

## S6 Table. All-cause and cause-specific total hospital admission rate* (per year) among in-center HD patients, by dialysis schedule (MWF or TTS), day of the week, and primary cause of admission

|  | MWF | |  | TTS | |
| --- | --- | --- | --- | --- | --- |
| Day | Number of Events | Rate (95% CI)  (per year) |  | Number of Events | Rate (95% CI)  (per year) |
| *All-cause hospital admission* | | | | | |
| Sun | 16,974 | 1.24 (1.22,1.26) |  | 12,698 | 1.12 (1.10,1.14) |
| Mon | 32,575 | 2.37 (2.35,2.40) |  | 24,153 | 2.14 (2.11,2.16) |
| Tue | 26,538 | 1.94 (1.92,1.96) |  | 25,097 | 2.23 (2.20,2.26) |
| Wed | 26,115 | 1.90 (1.87,1.92) |  | 20,308 | 1.79 (1.76,1.81) |
| Thu | 22,573 | 1.64 (1.62,1.66) |  | 20,471 | 1.81 (1.78,1.83) |
| Fri | 24,625 | 1.79 (1.77,1.81) |  | 18,482 | 1.63 (1.61,1.65) |
| Sat | 15,500 | 1.13 (1.11,1.15) |  | 15,565 | 1.38 (1.35,1.40) |
| *Cardiovascular-related admission* | | | | | |
| Sun | 4,583 | 0.33 (0.32,0.34) |  | 3,308 | 0.29 (0.28,0.30) |
| Mon | 8,738 | 0.64 (0.62,0.65) |  | 6,482 | 0.57 (0.56,0.59) |
| Tue | 6,596 | 0.48 (0.47,0.49) |  | 6,548 | 0.58 (0.57,0.59) |
| Wed | 6,464 | 0.47 (0.46,0.48) |  | 5,049 | 0.44 (0.43,0.46) |
| Thu | 5,474 | 0.40 (0.39,0.41) |  | 4,993 | 0.44 (0.43,0.45) |
| Fri | 5,968 | 0.43 (0.42,0.44) |  | 4,382 | 0.39 (0.37,0.40) |
| Sat | 3,567 | 0.26 (0.25,0.27) |  | 3,602 | 0.32 (0.31,0.33) |
| *Infection-related admission* | | | | | |
| Sun | 4,140 | 0.30 (0.29,0.31) |  | 3,023 | 0.27 (0.26,0.28) |
| Mon | 7,731 | 0.56 (0.55,0.58) |  | 5,489 | 0.49 (0.47,0.50) |
| Tue | 6,093 | 0.45 (0.43,0.46) |  | 5,837 | 0.52 (0.50,0.53) |
| Wed | 6,479 | 0.47 (0.46,0.48) |  | 4,763 | 0.42 (0.41,0.43) |
| Thu | 5,252 | 0.38 (0.37,0.39) |  | 4,948 | 0.44 (0.42,0.45) |
| Fri | 6,263 | 0.46 (0.44,0.47) |  | 4,528 | 0.40 (0.39,0.41) |
| Sat | 4,053 | 0.30 (0.29,0.30) |  | 4,132 | 0.37 (0.35,0.38) |
| *Vascular access-related admission* | | | | | |
| Sun | 784 | 0.06 (0.05,0.06) |  | 651 | 0.06 (0.05,0.06) |
| Mon | 2,143 | 0.16 (0.15,0.17) |  | 1,577 | 0.14 (0.13,0.15) |
| Tue | 1,866 | 0.14 (0.13,0.14) |  | 1,626 | 0.15 (0.14,0.16) |
| Wed | 1,879 | 0.14 (0.13,0.14) |  | 1,449 | 0.13 (0.12,0.14) |
| Thu | 1,583 | 0.12 (0.11,0.12) |  | 1,402 | 0.13 (0.12,0.13) |
| Fri | 1,911 | 0.14 (0.13,0.15) |  | 1,326 | 0.12 (0.11,0.12) |
| Sat | 1,034 | 0.08 (0.07,0.08) |  | 1,232 | 0.11 (0.10,0.11) |

*Each rate was computed as the number of hospital admissions during follow-up in a group, divided by the amount of person-*years* at risk of hospitalization in that group; thus, the unit of each rate is ‘per *year*.’
