# Supplementary material for: Emergency department visits and hospitalizations among hemodialysis patients by day of the week and dialysis schedule in the United States
Source: PLoS One. 2019 Aug 15;14(8):e0220966. doi: 10.1371/journal.pone.0220966 (PMC6695146; doi:10.1371/journal.pone.0220966)
Supplement: S7 Table — (DOCX) [file pone.0220966.s007.docx]

## S7 Table. All-cause and cause-specific hospital admission not preceded by an ED visit rate* (per year) among in-center HD patients, by dialysis schedule (MWF or TTS), day of the week, and primary cause of admission

|  | MWF | |  | TTS | |
| --- | --- | --- | --- | --- | --- |
| Day | Number of Events | Rate (95% CI)  (per year) |  | Number of Events | Rate (95% CI)  (per year) |
| *All-cause hospital admission* | | | | | |
| Sun | 2,159 | 0.16 (0.15,0.16) |  | 1,663 | 0.15 (0.14,0.15) |
| Mon | 7,010 | 0.51 (0.50,0.52) |  | 5,743 | 0.51 (0.50,0.52) |
| Tue | 8,368 | 0.61 (0.60,0.63) |  | 6,193 | 0.55 (0.54,0.56) |
| Wed | 6,891 | 0.50 (0.49,0.51) |  | 5,886 | 0.52 (0.51,0.53) |
| Thu | 7,362 | 0.54 (0.52,0.55) |  | 5,412 | 0.48 (0.46,0.49) |
| Fri | 6,248 | 0.45 (0.44,0.47) |  | 5,232 | 0.46 (0.45,0.47) |
| Sat | 2,471 | 0.18 (0.17,0.19) |  | 2,140 | 0.19 (0.18,0.20) |
| *Cardiovascular-related admission* | | | | | |
| Sun | 569 | 0.04 (0.04,0.04) |  | 476 | 0.04 (0.04,0.05) |
| Mon | 1,795 | 0.13 (0.12,0.14) |  | 1,436 | 0.13 (0.12,0.13) |
| Tue | 2,089 | 0.15 (0.15,0.16) |  | 1,485 | 0.13 (0.13,0.14) |
| Wed | 1,638 | 0.12 (0.11,0.12) |  | 1,419 | 0.12 (0.12,0.13) |
| Thu | 1,772 | 0.13 (0.12,0.13) |  | 1,322 | 0.12 (0.11,0.12) |
| Fri | 1,371 | 0.10 (0.09,0.10) |  | 1,167 | 0.10 (0.10,0.11) |
| Sat | 512 | 0.04 (0.03,0.04) |  | 439 | 0.04 (0.04,0.04) |
| *Infection-related admission* | | | | | |
| Sun | 451 | 0.03 (0.03,0.04) |  | 333 | 0.03 (0.03,0.03) |
| Mon | 1,417 | 0.10 (0.10,0.11) |  | 1,029 | 0.09 (0.09,0.10) |
| Tue | 1,514 | 0.11 (0.11,0.12) |  | 1,140 | 0.10 (0.10,0.11) |
| Wed | 1,359 | 0.10 (0.09,0.10) |  | 1,110 | 0.10 (0.09,0.10) |
| Thu | 1,363 | 0.10 (0.09,0.10) |  | 972 | 0.09 (0.08,0.09) |
| Fri | 1,324 | 0.10 (0.09,0.10) |  | 1,047 | 0.09 (0.09,0.10) |
| Sat | 548 | 0.04 (0.04,0.04) |  | 480 | 0.04 (0.04,0.05) |
| *Vascular access-related admission* | | | | | |
| Sun | 127 | 0.01 (0.01,0.01) |  | 106 | 0.01 (0.01,0.01) |
| Mon | 703 | 0.05 (0.05,0.06) |  | 595 | 0.05 (0.05,0.06) |
| Tue | 852 | 0.06 (0.06,0.07) |  | 615 | 0.05 (0.05,0.06) |
| Wed | 718 | 0.05 (0.05,0.06) |  | 593 | 0.05 (0.05,0.06) |
| Thu | 777 | 0.06 (0.05,0.06) |  | 530 | 0.05 (0.04,0.05) |
| Fri | 699 | 0.05 (0.05,0.05) |  | 529 | 0.05 (0.04,0.05) |
| Sat | 198 | 0.01 (0.01,0.02) |  | 221 | 0.02 (0.02,0.02) |

*Each rate was computed as the number of hospital admissions during follow-up in a group, divided by the amount of person-*years* at risk of hospitalization in that group; thus, the unit of each rate is ‘per *year*.’
