# Supplementary material for: Emergency department visits and hospitalizations among hemodialysis patients by day of the week and dialysis schedule in the United States
Source: PLoS One. 2019 Aug 15;14(8):e0220966. doi: 10.1371/journal.pone.0220966 (PMC6695146; doi:10.1371/journal.pone.0220966)
Supplement: S4 Fig — Adjusted incidence rate (per 100/year)* of cause-specific ED visits not followed by a hospital admission for in-center HD patients in 2013, by dialysis schedule ((a) MWF vs. (b) TTS), day of the week and primary diagnosis of ED visits. (DOCX) [file pone.0220966.s011.docx]

## S4 Fig. Adjusted incidence rate (per 100/year)* of cause-specific ED visits not followed by a hospital admission for in-center HD patients in 2013, by dialysis schedule ((a) MWF vs. (b) TTS), day of the week and primary diagnosis of ED visits

*Incidence rates are adjusted for age, sex, race/ethnicity, the Charlson Comorbidity Index score, HD vintage, HD session length (in minutes), Kt/V, IDWG, and BMI
